# Bioprinted Micro‐Clots for Kinetic Analysis of Endothelial Cell‐Mediated Fibrinolysis

**DOI:** 10.1002/adhm.202403043

**Published:** 2025-01-31

**Authors:** Jonathan J. Chang, Kelsey Brew, Jamie A.G. Hamilton, Varun Kumar, José A. Diaz, Shuichi Takayama

**Affiliations:** ^1^ Wallace H. Coulter Department of Biomedical Engineering Georgia Institute of Technology and Emory University Atlanta GA 30332 USA; ^2^ The Parker H. Petit Institute of Bioengineering and Bioscience Georgia Institute of Technology Atlanta GA 30332 USA; ^3^ Division of Surgical Research Vanderbilt University Medical Center Nashville TN 37232 USA

**Keywords:** endothelial cells, fibrinolysis, micro‐physiological systems, thrombosis

## Abstract

Vascular hypo‐fibrinolysis is a historically underappreciated and understudied aspect of venous thromboembolism (VTE). This paper describes the development of a micro‐clot dissolution assay for quantifying the fibrinolytic capacity of endothelial cells – a key driver of VTE development. This assay is enabled using aqueous two‐phase systems (ATPS) to bioprint microscale fibrin clots over human umbilical vein endothelial cells (HUVECs). Importantly, these micro‐clots are orders of magnitude smaller than conventional fibrin constructs and allow HUVEC‐produced plasminogen activators to mediate visually quantifiable fibrinolysis. Using live‐cell time‐lapse imaging, micro‐clot dissolution by HUVECs is tracked, and fibrinolysis kinetics are quantified. The sensitivity of cell‐driven fibrinolysis to various stimuli is rapidly tested. The physiological relevance of this convenient high‐throughput assay is illustrated through treatments with lipopolysaccharide (LPS) and rosuvastatin that elicit anti‐ and pro‐fibrinolytic responses, respectively. Furthermore, treatment with baricitinib, an anti‐inflammatory therapeutic found to increase cardiovascular risks after market approval, provokes an anti‐fibrinolytic response – which highlights the potential role of endothelial cells in increasing VTE risk for patients receiving this drug. This endothelial cell fibrinolysis assay provides a high‐throughput and versatile drug testing platform – potentially allowing for early preclinical identification of therapeutics that may beneficially enhance or adversely impair endothelial fibrinolysis.

## Graphical abstract

This paper describes the development of a microscale model that quantifies the breakdown of fibrin by endothelial cells. The small size scale of this engineered system enables fibrin breakdown to be observed without the addition of external activators. This system could help in identifying compounds which both promote and hinder thrombosis.

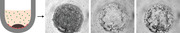

## 1 Introduction

In response to vascular injury, fibrin‐rich clots are rapidly formed to prevent excessive blood loss. This process is triggered by exposure of the subendothelial matrix and activation of the extrinsic coagulation pathway.^[^ ^1^ ^]^ In the final steps of coagulation, thrombin converts fibrinogen into fibrin to form a polymer mesh that holds the clot together.^[^ ^2^ ^]^ Fibrinolysis – the enzymatic degradation of fibrin – is an opposing process to coagulation and is critical to clot dissolution.^[^ ^3^ ^]^ Under pathological conditions, vascular clots can resist dissolution and propagate, leading to venous thromboembolism (VTE)^[^ ^4^ ^]^ – the inappropriate formation of occluding blood clots within veins. VTE refers to both deep vein thrombosis (DVT) and pulmonary embolism (PE) and results in an estimated 500000 deaths annually in the United States.^[^ ^5^ ^]^ Novel treatment strategies are greatly needed to help improve patient outcomes.

Historically, VTE research has focused predominantly on hypercoagulability.^[^ ^6^ ^]^ As a result, VTE treatment today centers around the use of anticoagulants.^[^ ^5^, ^7^ ^]^ These treatment strategies inhibit the formation and propagation of venous clots, but they do not directly enhance clot dissolution.^[^ ^4^ ^]^ In the past two decades, hypo‐fibrinolysis has emerged as an important risk factor for VTE development,^[^ ^6^ ^]^ and pre‐clinical studies with novel pro‐fibrinolytic therapies show promise for improving VTE patient care.^[^ ^8^ ^]^ Currently, thrombolytics (i.e., exogenous plasminogen activators) are the only clinically utilized therapies that target fibrinolysis but are limited in their use due to an increased risk of major bleeding.^[^ ^9^ ^]^ Therefore, the development of VTE therapeutics which more precisely regulate fibrinolytic capacity remains an area of research need.

Due to the biological complexity of venous thrombosis, pre‐clinical development of therapies relies heavily on the use of animal models.^[^ ^10^ ^]^ Bioengineered systems mimicking in vivo conditions are being developed, but these models often require labor‐intensive microfluidic setups and tend to focus on coagulation instead of fibrinolysis.^[^ ^11^ ^]^ Clinically, several in vitro assays exist to assess fibrinolysis. In the majority of these methods, patient blood samples are clotted, plasminogen activators are added, and fibrinolysis is tracked through turbidimetric changes.^[^ ^12^, ^13^ ^]^ While these systems are beneficial for diagnosing fibrinolytic disorders and can be used to evaluate thrombolytics that directly induce clot lysis, their simplicity precludes mechanistic studies on how different factors can exacerbate or impede VTE development. Notably, these clinical clot lysis assays mostly neglect cellular contributions to fibrinolysis,^[^ ^12^, ^13^ ^]^ and rarely include endothelial cells. Endothelial cell phenotype is a driver of VTE development.^[^ ^14^ ^]^ In Virchow's Triad, the prevailing theory on factors that contribute to thrombosis, endothelial damage/dysfunction makes up one of the three central pillars.^[^ ^15^ ^]^ Moreover, endothelial cells are capable of controlling thrombosis and fibrinolysis through their production of plasminogen activator inhibitor‐1 (PAI‐1) and tissue‐type plasminogen (tPA).^[^ ^1^, ^3^ ^]^

**Figure 1:**
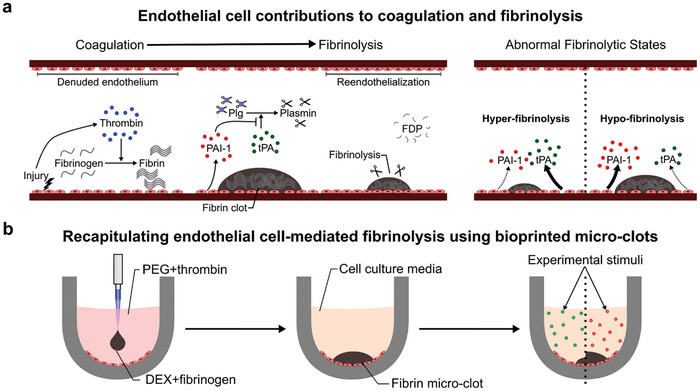
Recapitulating endothelial cell‐mediated fibrinolysis using bioprinted micro‐clots. a) Left: In response to vascular injury, the extrinsic coagulation cascade is triggered leading to the thrombin‐mediated conversion of fibrinogen to a fibrin clot. As the tissue heals, the breakdown of these clots is carefully regulated by a host of factors with key contributors being PAI‐1 and tPA. Plasminogen (Plg) is converted to plasmin, the active fibrinolytic enzyme, by tPA, and this process is inhibited by PAI‐1. Right: In pathological states, the control of fibrinolysis is dysregulated. Hyper‐fibrinolysis can lead to severe bleeding due to the premature removal of the clot. Hypo‐fibrinolysis can lead to clot persistence and thrombosis. b) We recreate this physiological process by seeding HUVECs in a 96‐well U‐bottom plate. A PEG/DEX ATPS is utilized to separate thrombin and fibrinogen in each phase respectively, thereby allowing for the controlled formation of the fibrin micro‐clot. Over time, endothelial cell‐produced factors lead to the dissolution of the micro‐clot. Using this platform, the impacts of various experimental stimuli on the fibrinolytic balance can be quantified.

For these reasons, we focused on developing an in vitro micro‐clot dissolution assay that directly evaluated endothelial cell contributions to fibrinolysis. Adapting our previously established fibrin bioprinting technology,^[^ ^16^, ^17^ ^]^ we formed microscale clots directly over primary human umbilical vein endothelial cells (HUVEC) and observed clot dissolution over time using live‐cell imaging. This system mimics a physiological situation where a fibrin clot forms over injured endothelial cells and begins to degrade as the tissue heals. We then applied the model to probe how different factors alter the fibrinolytic balance (**Figure** **1**). This assay provides a high‐throughput and versatile platform that may be particularly useful in hypothesis generation experiments where potential VTE exacerbating factors and therapeutics may be identified.

## 2 Results

### 2.1 Micro‐Clot Formation Using ATPS and Real‐Time Observation of Fibrinolysis

**Figure 2:**
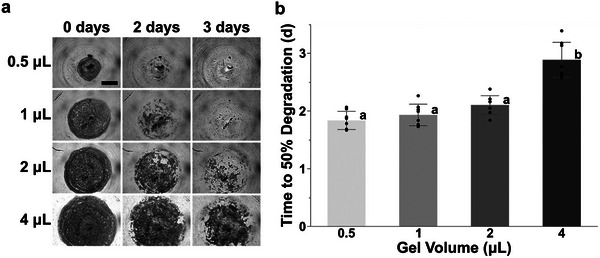
Evaluating differences in the time to visualize fibrinolysis of various‐sized fibrin micro‐clots. a) Representative images showing the degradation of different‐sized micro‐clots over the course of 3 days. Scale bar is 1 mm. b) Time to visualize fibrinolysis increases non‐linearly with micro‐clot volume. Data is shown as mean ± standard deviation. Conditions not connected by the same letter are statistically significant (*p* < 0.05 by ANOVA with post‐hoc Tukey's test). *N* = 8 for all conditions.

To physiologically recapitulate in vivo conditions during endothelial injury and early fibrin clot formation, we start with a proliferating HUVEC layer – shown previously to upregulate pathways associated with wound healing and injury.^[^ ^18^, ^19^ ^]^ Then, we replicate the final steps of the coagulation cascade (i.e., thrombin‐induced conversion of fibrinogen to fibrin) using ATPS bioprinting to form micro‐clots directly over the HUVECs (Figure 1). Fibrin micro‐clots are maintained for up to 7 days in fully supplemented VCBM with 10% FBS. FBS serves as a source of plasminogen which is normally produced in the liver and enhances the rate of fibrinolysis (Figure S4, Supporting Information). Degradation of these micro‐clots can be tracked using an Incucyte imager. Using this methodology, we rapidly form microliter and sub‐microliter‐sized fibrin clots in a 96‐well plate (**Figure** **2**a). Notably, we observe a non‐linear increase in fibrinolysis time with increasing micro‐clot size (Figure 2b). In this experiment, cell density and FBS amount are fixed. Consequently, the larger‐sized clots would be subjected to relatively reduced cell numbers and plasminogen levels. This highlights the importance of the small volumes used in this assay; larger volumes would substantially increase fibrinolysis times without the addition of exogenous activators.

Additionally, we aimed to characterize endothelial cell health before, during, and after micro‐clot degradation. Using an LDH‐Glo assay, we determined that cytotoxicity levels remained low (less than 25%) for all timepoints examined (Figure S5, Supporting Information). When pre‐treated with LPS, cytotoxicity levels did increase slightly. LPS pre‐treatment could be an effective approach to examining micro‐clot degradation in an inflammatory state.

### 2.2 Micro‐Clot Dissolution is Controlled by HUVECs and can be Attenuated by PAI‐1

**Figure 3:**
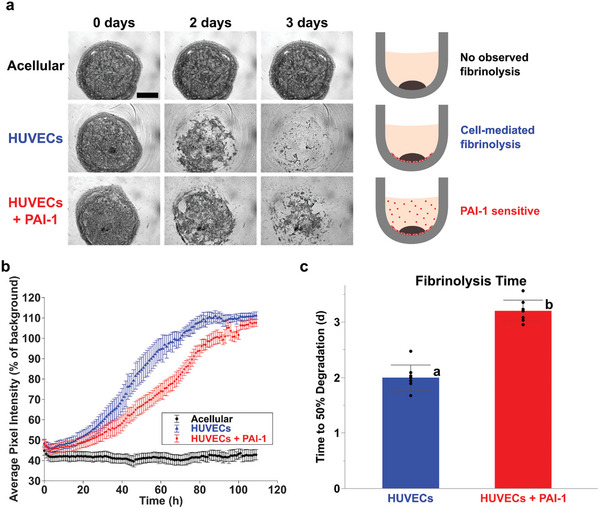
a) Fibrin micro‐clots printed in the absence of cells (acellular) show no visual degradation over time, while clots printed over HUVECs degrade over the course of several days. Fibrinolysis can be attenuated by the addition of 2.5 µg mL^−1^ PAI‐1. Scale bar is 1 mm. b) Changes in average pixel intensity of the fibrin region over time can be quantified with higher values corresponding to a greater degree of fibrinolysis. c) PAI‐1 significantly increases fibrinolysis time. Data is shown as mean ± standard deviation. *N* = 8 for all conditions.

With our micro‐clot dissolution assay, we aimed to investigate endothelial cell‐mediated fibrinolysis. To rule out potential fibrinolytic contributions from the media and serum, we compared micro‐clot dissolution with and without (i.e., acellular) HUVECs present. Moreover, PAI‐1 addition (2.5 µg mL^−1^) was investigated to confirm that the HUVECs were achieving fibrinolysis by secretion of plasminogen activators (**Figure** **3**a). This supraphysiological concentration of PAI‐1 was used due to the short half‐life of PAI‐1. In the acellular condition, no fibrinolysis (i.e., no change in pixel intensity) was observed over the course of the entire experiment. However, for both HUVEC‐containing conditions, a sigmoidal fibrinolysis profile is observed (Figure 3b); both degradation profiles plateaued at a similar value indicating the complete dissolution of the micro‐clots. However, the PAI‐1 treated condition took longer to reach this plateau due to the decreased rate of fibrinolysis. These results showcase this assay's ability to capture endothelial cell‐mediated fibrinolysis. The slow start to fibrinolysis may reflect the known effect of thrombin to stimulate increased tPA production from HUVECs after an initial lag period.^[^ ^20^ ^]^ Fibrinolysis time or the time to 50% degradation increased significantly when PAI‐1 was added to the assay, thereby confirming our assay's sensitivity to the fibrinolytic inhibitor (Figure 3c).

### 2.3 LPS Slows Endothelial Cell‐Mediated Fibrinolysis

**Figure 4:**
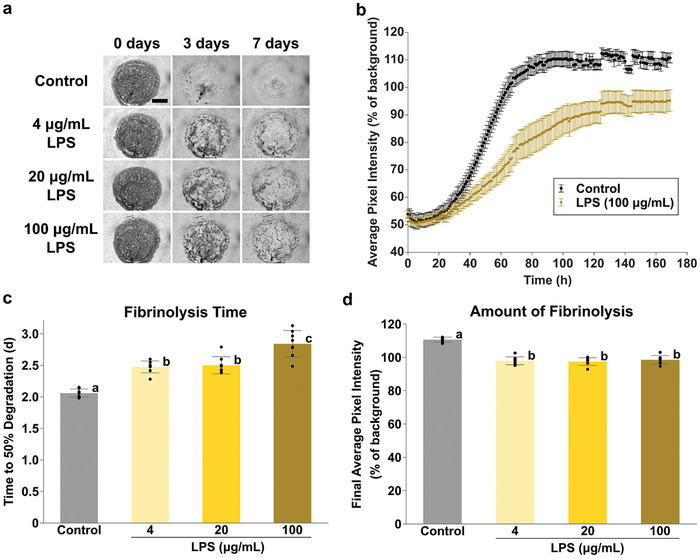
LPS attenuates the speed and extent of endothelial cell‐mediated fibrinolysis. a) Representative images showing the degradation of micro‐clots under various LPS concentrations over the span of 1 week. Scale bar is 1 mm. b) Pixel intensity time course plots comparing the fibrinolysis of non‐treated and LPS‐treated (100 µg mL^−1^) endothelial cells. c,d) Fibrinolysis time and amount of fibrinolysis at increasing LPS concentrations. All data is shown as mean ± standard deviation. Conditions not connected by the same letter are statistically significant (*p* < 0.05 by ANOVA with post‐hoc Tukey's test). *N* = 8 for all conditions.

Bacterial LPS is known to promote thrombosis in vivo,^[^ ^21^, ^22^ ^]^ and human endothelial cells treated with LPS in vitro increase expression and synthesis of plasminogen activator inhibitors.^[^ ^22^, ^23^ ^]^ Therefore, we aimed to determine the sensitivity of our assay to LPS. LPS was tested at three different concentrations in our system, and clot dissolution was monitored over the course of 7 days. Qualitatively, the time‐lapse imaging showed that LPS slows fibrinolysis at all three concentrations tested, and that residual fibrin remains undegraded at the end of the experiment (**Figure** **4**a). Plotting the change in average pixel intensity for the fibrin region over time corroborates these results; LPS flattens the fibrinolysis profile in comparison to the untreated control (Figure 4b). All 3 LPS concentrations cause 1) a significant increase in the time to 50% degradation with 100 µg mL^−1^ LPS having the greatest effect (Figure 4c) and 2) a significant decrease in the amount of fibrinolysis (i.e., the upper asymptote of the logistic curve) (Figure 4d). Furthermore, the concentrations of LPS tested do not significantly affect cell viability, thus verifying that the attenuation of fibrinolysis cannot be attributed to extensive cell death (Figure S6, Supporting Information). This platform captures the hypo‐fibrinolytic state of HUVECs induced by LPS and has potential utility as a disease model of inflammation‐induced hypo‐fibrinolysis.

### 2.4 Investigation of Pro‐ and Anti‐Fibrinolytic Therapeutics

**Figure 5:**
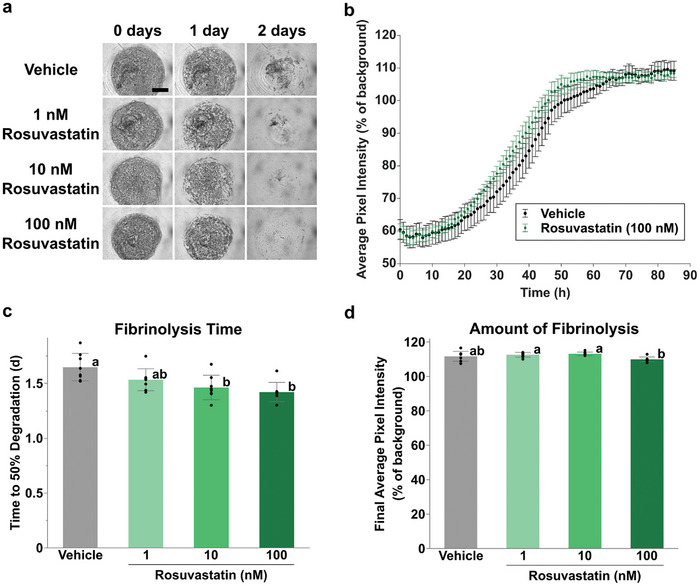
Rosuvastatin enhances endothelial cell‐mediated fibrinolysis. a) Representative images showing the degradation of micro‐clots under various rosuvastatin concentrations over the span of 2 days. Scale bar is 1 mm. b) Pixel intensity time course plots comparing the fibrinolysis of vehicle‐treated and rosuvastatin‐treated (100 nm) endothelial cells. c,d) Fibrinolysis time and amount of fibrinolysis at increasing rosuvastatin concentrations. All data is shown as mean ± standard deviation. Conditions not connected by the same letter are statistically significant (*p* < 0.05 by ANOVA with post‐hoc Tukey's test). *N* = 8 for all conditions.

As a proof‐of‐concept, we examined the effects of drugs with known positive and negative effects on fibrinolysis. First, we investigated rosuvastatin – a drug that has shown promising pro‐fibrinolytic effects and the ability to reduce the risk of VTE in humans.^[^ ^24^, ^25^, ^26^, ^27^ ^]^ Statins in general have also been proposed as a possible prophylactic treatment for VTE.^[^ ^28^ ^]^ Rosuvastatin was tested at three different concentrations in our micro‐clot dissolution assay (**Figure** **5**). The analysis of the time‐lapse images revealed a dose‐dependent acceleration in fibrinolysis; 1 nm rosuvastatin was not found to have an effect on fibrinolysis, but 10 and 100 nm rosuvastatin concentrations significantly decreased the time to 50% degradation (Figure 5c). None of the drug concentrations tested significantly affected the final amount of fibrinolysis in comparison to the vehicle control (Figure 5d), thus indicating total clot dissolution under all rosuvastatin concentrations. Additionally, rosuvastatin treatment in the absence of endothelial cells was not seen to have any effect on fibrinolysis (Figure S7, Supporting Information), suggesting that rosuvastatin is acting on endothelial cells to enhance fibrinolysis.

**Figure 6:**
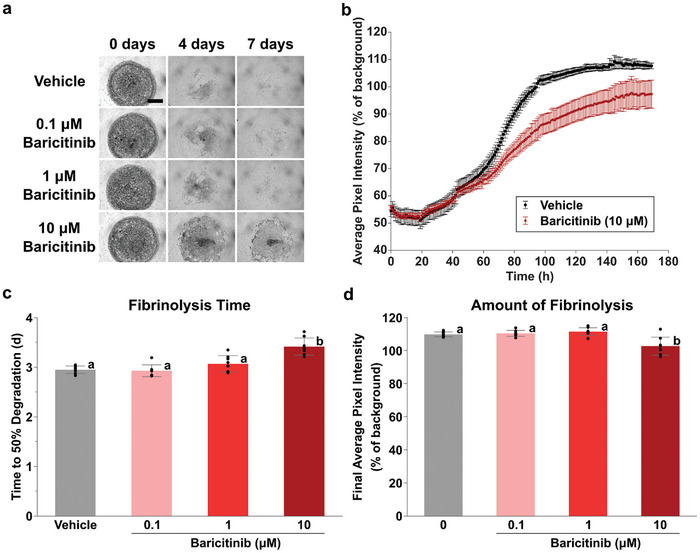
Baricitinib treatment hinders endothelial cell‐mediated fibrinolysis. a) Representative images showing the degradation of micro‐clots under various baricitinib concentrations over the span of 1 week. Scale bar is 1 mm. b) Pixel intensity time course plots comparing the fibrinolysis of vehicle‐treated and baricitinib‐treated (10 µm) endothelial cells. c,d) Fibrinolysis time and amount of fibrinolysis at increasing baricitinib concentrations. All data is shown as mean ± standard deviation. Conditions not connected by the same letter are statistically significant (*p* < 0.05 by ANOVA with post‐hoc Tukey's test). *N* = 8 for all conditions.

Next, we tested baricitinib – a JAK inhibitor used to treat COVID‐19, rheumatoid arthritis, and alopecia areata – in our assay. Baricitinib was of particular interest due to the recent FDA warnings of the increased risk of blood clots when using baricitinib or other JAK inhibitors. We treated HUVECs within our system with three concentrations of baricitinib (**Figure** **6**). After running the clot dissolution assay for 7 days, the highest concentration of baricitinib tested (i.e., 10 µm) attenuated fibrinolysis and left residual fibrin undegraded as indicated by the flattened degradation profile (Figure 6a,b). Quantitative image analysis showed that the 10 µm baricitinib concentration significantly increased time to 50% degradation and decreased the final amount of fibrinolysis (Figure 6c,d). Notably, the 10 µm baricitinib condition did slightly decrease cell viability which may account for some of the observed effects. However, cell viability remained above 90% with this treatment (Figure S8, Supporting Information). Previous studies have suggested that baricitinib and other JAK inhibitors may increase the risk of thrombosis in part due to the activation of endothelial cells^[^ ^29^ ^]^ – which is supported by the results of this study.

Finally, aspirin – a widely used drug for reducing thrombosis risk – was tested. Aspirin is most known for its ability to lower thrombosis risk by reducing coagulation and impairing platelet activation/aggregation.^[^ ^30^, ^31^ ^]^ Aside from these effects, the impact of aspirin treatment on fibrinolysis remains controversial despite decades of research. Here, we use our system to specifically examine the effect of aspirin on endothelial cell‐mediated fibrinolysis after a clot is already formed and where no exogenous tPA is added. No significant effects on endothelial cell‐mediated fibrinolysis were seen with aspirin treatment (Figure S9, Supporting Information).

### 2.5 Evaluating Drug Responses Under Inflammatory Conditions

To examine the effect of drugs under inflammatory conditions, HUVECs were pre‐treated with LPS before micro‐clot printing. Then, either rosuvastatin or baricitinib was applied for the duration of the experiment. All concentrations of rosuvastatin tested did not significantly alter fibrinolysis. The effect of rosuvastatin was insufficient to overcome the inflammatory conditions. For the baricitinib treatment under inflammatory conditions, the lower concentrations of baricitinib (i.e., 0.1 and 1 µm) significantly increased fibrinolysis in comparison to the LPS control. Interestingly, these results differ from the baricitinib results under normal conditions where these concentrations did not significantly impact fibrinolysis. The highest concentration of baricitinib (10 µm) still significantly hindered fibrinolysis under normal and inflammatory conditions (Figure S10, Supporting Information). Future studies can expand on this work through more extensive testing of various drugs and conditions.

## 3 Discussion and Conclusion

Historically, the role of the fibrinolytic system in DVT and PE was overshadowed by the importance of the coagulation system, which forms the thrombus. Yet more recently, mouse models have demonstrated the critical role of the fibrinolytic system in influencing thrombus size.^[^ ^32^ ^]^ Using a well‐established ligation model, we previously showed that thrombus size was significantly increased or decreased when using apolipoprotein E knockout (hypo‐fibrinolytic) or PAI‐1 knockout (hyper‐fibrinolytic) mice respectively.^[^ ^32^ ^]^ These results indicated that the coagulation system cannot form clots at its maximum expression due to the contrasting action of the fibrinolytic system. In VTE pathogenesis, fibrinolysis is impaired leading to the less restrained formation of clots.

Due to its critical role in VTE pathogenesis, the fibrinolytic system has emerged as a promising therapeutic target. However, recombinant tPA is currently the only FDA‐approved thrombolytic treatment, and concerns over bleeding risk preclude its widespread use. New fibrinolysis‐modulating drugs are being developed to combat VTE and a wide array of other fibrinolysis‐related diseases.^[^ ^33^ ^]^ Therefore, more physiologically relevant disease models of clot dissolution, such as the one developed here, will be critical to support these advances.

### 3.1 A plasminogen Activator‐Free Fibrinolysis Assay

In general, traditional fibrinolysis assays require the addition of excess exogenous plasminogen activators to initiate fibrinolysis.^[^ ^12^ ^]^ With the added plasminogen activators, complete clot dissolution occurs in a matter of minutes. While these assays have clinical utility, they are insufficient for mechanistic studies that probe cellular contributions to fibrinolysis. The rapid dissolution of clots promoted by the exogenous plasminogen activators would mask cellular effects which require longer timescales to manifest. In fact, many of the traditional fibrinolysis assays are acellular – aside from those that utilize whole blood samples. Here, we have developed a more physiologically relevant assay that is 1) free of exogenous plasminogen activators and 2) relies on endothelial cell‐produced fibrinolytic factors. Notably, we successfully quantified the fibrinolytic capacity of HUVECs which was previously challenging due to the low tPA production of these cells.^[^ ^34^ ^]^

Plasminogen and tPA are known to have poor penetration into clots, and thus, mainly act on the clot surface.^[^ ^35^ ^]^ As clot size decreases, the surface area to volume ratio increases exponentially. By utilizing microliter scale fibrin clots, we hypothesized that the favorable surface area to volume ratios would provide a proportionally larger surface for fibrinolysis to occur, thereby enhancing the effect of cell‐produced fibrinolytic factors. This hypothesis was corroborated experimentally, and we showed a strictly increasing and convex relationship between fibrinolysis time and clot volume. Importantly, plasminogen activator‐free clot dissolution was achieved by using fibrin clots that were 2–3 orders of magnitude smaller in volume compared to traditional assays.

### 3.2 Comprehensive and Real‐Time Measurement of Fibrinolytic State

By directly visualizing micro‐clot dissolution using automated live‐cell imaging, we obtain a more comprehensive measurement of the fibrinolytic state. While endpoint measurements of tPA or PAI‐1 can provide some insight, these metrics in isolation fail to provide a holistic picture of the fibrinolytic state. In addition to tPA and PAI‐1, a multitude of other elements can impact whether fibrinolysis occurs.^[^ ^3^ ^]^ Moreover, many of the fibrinolytic components have short half‐lives on the order of minutes,^[^ ^3^, ^36^ ^]^ which can obscure and hinder ex vivo measurements of their activity. Our system inherently accounts for the contributions of all present components to more accurately assess the fibrinolytic state in real‐time. The endothelial cells in our assay also provide a physiologically relevant context to assess fibrinolysis as the endothelial surface can bind both tPA and plasminogen to accelerate plasmin generation more than 60‐fold.^[^ ^37^ ^]^

The results of our aspirin experimentation demonstrate the benefits of this comprehensive metric of fibrinolytic state. Previous studies have identified two possible mechanisms through which aspirin accelerates fibrinolysis: 1) aspirin acetylates fibrinogen, leading to the formation of more permeable clots which are more easily dissolved^[^ ^31^, ^38^ ^]^ and 2) aspirin increases endothelial cell‐produced tPA.^[^ ^31^ ^]^ While the first mechanism has been well‐established, the second remains contested with different studies reporting increased, decreased, or unaltered tPA levels and activity.^[^ ^31^ ^]^ While our results do not directly measure tPA levels, we help to clarify the debate by showing that aspirin treatment does not meaningfully impact endothelial cell‐mediated fibrinolysis. Notably, the micro‐clots used in our assay are formed in the absence of aspirin, thereby allowing us to isolate the potential effects of aspirin on endothelial cells from its effects on clot permeability.

### 3.3 Assay Utility and Versatility in Drug Development and Testing

The incorporation of endothelial cells within our system enables novel biological insights for drug development studies. The jupiter trial demonstrated that rosuvastatin was associated with a decreased rate of DVT in 2009.^[^ ^27^ ^]^ The trial authors subsequently invited the research community to investigate why a non‐anticoagulant drug had such a positive effect on the DVT patient population. In 2013, we were the first to report on the pro‐fibrinolytic effect of rosuvastatin in DVT animal models.^[^ ^39^ ^]^ To build off this work, we examined the effects of rosuvastatin in our in vitro system. In the absence of endothelial cells, rosuvastatin failed to initiate any measurable degree of fibrinolysis. However, we showed an acceleration of fibrinolysis with rosuvastatin treatment in the presence of endothelial cells. These results suggest that rosuvastatin promotes clot dissolution through cell‐mediated mechanisms rather than direct fibrinolytic effects. The ability to decouple fibrinolytic effects presents more evidence of the utility of our assay in VTE drug development.

Another capability of our system that is distinct from traditional assays is the ability to investigate clot persistence. Traditional assays are heavily biased toward a pro‐fibrinolytic state and clot dissolution will always occur due to the addition of excess exogenous activators. However, this feature presents a challenge for investigating stimuli that promote a hypo‐fibrinolytic state. Our assay begins with a more neutral fibrinolytic balance and allows us to probe factors that shift the balance in both directions. Our LPS and baricitinib results demonstrate our ability to quantify hypo‐fibrinolytic states. Importantly, baricitinib comes from a class of drugs – JAK kinase inhibitors – whose thrombotic risk was not readily apparent until after the start of their use in clinical populations.^[^ ^40^ ^]^ The late discovery of thrombotic risks is a common occurrence in drug development, owing to a lack of reliable preclinical screening methodologies.^[^ ^41^ ^]^ Our high‐throughput and robust system could serve as a useful platform for the early identification of a drug's thrombotic risk.

### 3.4 Limitations and Future Directions

Our assay recapitulates a simplified clot composition made of solely fibrin. While these fibrin‐only clots successfully captured the effects of the stimuli tested in this manuscript, other biological questions may require a more complex clot composition and structure. Future studies could systematically examine the incorporation of additional cell types and clot components to measure their impacts on fibrinolysis. Additionally, the disturbed flow seen in thrombosis is absent from this system. Once the contributions of endothelial cells have been elucidated using this system, different flow conditions could be tested to investigate how they impact results. The bottom‐up engineering approach of our system would easily allow the combination of clot components to 1) replicate specific physiological and pathophysiological states and 2) answer increasingly more complex mechanistic questions related to clot dissolution and persistence.

Finally, endothelial cells play a key role in preventing thrombosis due to their involvement in the fibrinolytic process. Here, we present a phenotypic assay to evaluate endogenous endothelial cell fibrinolysis. We hope that this platform will generate novel biological insights and accelerate the development of fibrinolysis‐modulating treatments.

## 4 Experimental Section

### Cell Culture and Seeding

HUVECs were purchased from the American Type Culture Collection (ATCC, PCS‐100‐013, lot 70032759) and grown in vascular cell basal media (VCBM; ATCC, PCS‐100‐030) supplemented with an endothelial cell growth kit (ATCC, PCS‐100‐041) and 1% penicillin‐streptomycin (Gibco, #15070‐063). Cultures were maintained at 37 °C and 5% CO_2_ and passaged routinely at 70–80% confluency. Cells were not cultured past passage 8. For the assay, HUVECs were seeded 24 h prior to fibrin bioprinting at a density of 5000 cells/well in a 96‐well cell culture‐treated U‐bottom microplate (Corning, #353077).

### ATPS Bioprinting of Fibrin Micro‐Clots

A stock solution of 20% w/w DEX 500 kDa (Sigma, #31392) was prepared in phosphate‐buffered saline (PBS), and a stock solution of 6% w/w PEG 35 kDa (Sigma, #81310) was prepared in Dulbecco's Modified Eagle's Medium (DMEM; ATCC, #30‐2002) with 10% w/w distilled water. These DEX and PEG solutions were sterile filtered and stored at 4 °C.

Before bioprinting the fibrin micro‐clots, all reagents were warmed to 37 °C in a bead bath. A PEG‐thrombin solution was created by adding human α‐thrombin (Enzyme Research Laboratories, HT 1002a) to the PEG stock solution to achieve a final thrombin concentration of 0.5 U mL^−1^. To make the DEX‐fibrinogen solution, the stock DEX solution was diluted to 3% with 10x Minimum Essential Medium (MEM; Gibco, #11430‐030), distilled water, human fibrinogen (Enzyme Research Laboratories, FIB 3), and DMEM (ATCC, #30‐2002). The final concentrations of 10x MEM, fibrinogen, and DMEM were 4.4% v/v, 4 mg mL^−1^, and 50% v/v respectively.

Once the working ATPS solutions were prepared as described above, media was removed from each well of the HUVEC‐seeded microplate and replaced with the PEG‐thrombin solution. 2 µL (unless otherwise noted) of the DEX‐fibrinogen solution was then immediately added to each well using a repeater pipet (Eppendorf). The microplate was then incubated at 37 °C for 30 min to allow for polymerization of the fibrin micro‐clots. After incubation, the ATPS reagents were removed and replaced with fully supplemented VCBM containing 10% fetal bovine serum (FBS) and any stimuli of interest (Figure 1).

### Automated Live‐Cell Imaging and Analysis

After bioprinting, the fibrin micro‐clots, microplates were placed into the Incucyte S3 (Sartorius). Brightfield scans of each well were taken every hour for the duration of the experiment (up to 7 days) on the spheroid module using the 4x objective.

After completion of the assay, videos of the degrading micro‐clots were exported and analyzed in MATLAB. First, a mask image was created of the first frame in each video – isolating the fibrin region as the foreground of the image; the following steps describe the process used to generate the mask: 1) The first frame was binarized and inverted. 2) A morphological closing process was applied to remove noise. 3) The imfill function was utilized to fill holes in the mask. 4) A morphological opening process was applied to remove small objects from the image foreground. 5) The bwareafilt function was used to isolate the largest object in the image foreground – the fibrin region (Figure S1, Supporting Information).

Next, the generated masks were used to filter all other frames in their respective video. This filtering process separated the initial fibrin area in each image from the background. The mean pixel intensity of the initial fibrin region was then calculated for all frames. Due to changes in overall image brightness throughout the experiment duration, this value is reported as a percentage of the mean background intensity of each image – this value will be referred to as average pixel intensity (Equation 1). Changes in average pixel intensity over time were then fit to a 4‐parameter logistic curve to quantify fibrinolysis time and amount of fibrinolysis (Figure S2, Supporting Information). Curve fitting is utilized to provide a convenient and unbiased method for describing the degradation profile. Parameters c and d from the degradation profile equation were directly used as objective measurements of fibrinolysis. Parameter c measures the speed of fibrinolysis as it represents the time it takes for 50% degradation to occur. Parameter d measures the final amount of fibrinolysis as it represents the upper asymptote value of the degradation profile.

(1) $$\mathrm{Aver}\mathrm{age}\;\mathrm{Pixel}\;\mathrm{Inte}\mathrm{nsit}y_{\mathrm{Frame}=i}=\frac{\mathrm{Mean}\;\mathrm{Fibr}\mathrm{in}\;\mathrm{Regi}\mathrm{on}\;\mathrm{Inte}\mathrm{nsit}y_{i}}{\mathrm{Mean}\;\mathrm{Back}\mathrm{grou}\mathrm{nd}\;\mathrm{Inte}\mathrm{nsit}y_{i\;}}\;\times 100$$

After complete degradation of the fibrin micro‐clots, the average pixel intensity will reach a value above 100%; this is caused by the final images having a naturally brighter center (Figure S3, Supporting Information).

### PAI‐1 Testing

Recombinant human PAI‐1 (Sigma, A8111) was tested in the micro‐clot dissolution assay. For acellular conditions, no HUVECs were seeded in the 96‐well plate prior to fibrin bioprinting. The PAI‐1 protein was added to fully supplemented VCBM containing 10% FBS immediately prior to performing the assay and replaced every 48 h.

### Stimuli Preparation and Testing

Lipopolysaccharide (LPS; Sigma, L2630), rosuvastatin (Selleck Chemicals, S2169), aspirin (Selleck Chemicals, S3017), and baricitinib (Cayman Chemical, #16707), were all tested as stimuli in the micro‐clot dissolution assay. All stimuli were thawed and added to fully supplemented VCBM containing 10% FBS immediately prior to performing the assay. Serial dilution was used to achieve the lower concentrations of all stimuli. For the aspirin and baricitinib experiments, all conditions contained 1% dimethyl sulfoxide (DMSO) including the vehicle control. For the rosuvastatin experiments, all conditions contained 0.1% DMSO including the vehicle control. Media changes with the added stimuli were performed every 48 h.

For combined LPS and drug testing, cells were treated with 4 µg mL^−1^ LPS in media for 24 h after seeding. After this 24 h pre‐treatment period, all LPS was washed out, and the micro‐clots were printed using the standard protocol. From this point, media changes with only the drugs (i.e., rosuvastatin or baricitinib) were performed every 48 h.

### Cell Viability and Cytotoxicity Measurements

For LIVE/DEAD staining, HUVECs were plated at 5 000 cells/well in a 96‐well plate (Corning, #3596), and 24 h after seeding, the stimuli – as described above – were prepared and added to the plate. The entire plate was incubated at 37 °C for 24 h. A mammalian LIVE/DEAD staining kit was used to measure cell viability. Media in each well of the 96‐well plate was replaced with a 2 µm calcein AM and 4 µm ethidium homodimer‐1 solution in media. After 15 min, each well was imaged with the Incucyte using the green and red channels and the 4x objective. The Incucyte Basic Analyzer was used to count the number of live and dead cells per image.

Cytotoxicity was quantified at 4 time points during the assay progression: pre‐clot loading, after‐clot loading, during clot degradation (Day 3), and after clot degradation (Day 7). Each sample consisted of 2.5 µL of media taken directly from the wells of the microplate. These samples were used in a lactate dehydrogenase (LDH) assay (Promega, #J2380) per manufacturer instructions. Each sample was made into a 100x dilution with LDH Storage Buffer, and each plate had luminescence recorded at 60 min according to manufacturer instructions. Technical Manual Version: Revised 7/23, TM548 LDH‐Glo Cytotoxicity Assay.

### Statistical Analysis

All statistical tests were performed using JMP with a significance level of 0.05. Results were displayed as mean ± standard deviation.

## Conflict of Interest

The authors declare no conflict of interest.

## Supporting information



Supporting Information

## Data Availability

Data Availability Statement: The datasets generated and/or analyzed in this study are available from the corresponding author upon reasonable request. The code used in this project is available at https://github.com/GT‐Shulab/.
