# Supplementary material for: Bioprinted Micro‐Clots for Kinetic Analysis of Endothelial Cell‐Mediated Fibrinolysis
Source: Adv Healthc Mater. 2025 Jan 31;14(7):2403043. doi: 10.1002/adhm.202403043 (PMC11912099; doi:10.1002/adhm.202403043)
Supplement: Supplementary file 1 — Supporting Information [file ADHM-14-0-s001.docx]

# Supplementary Materials


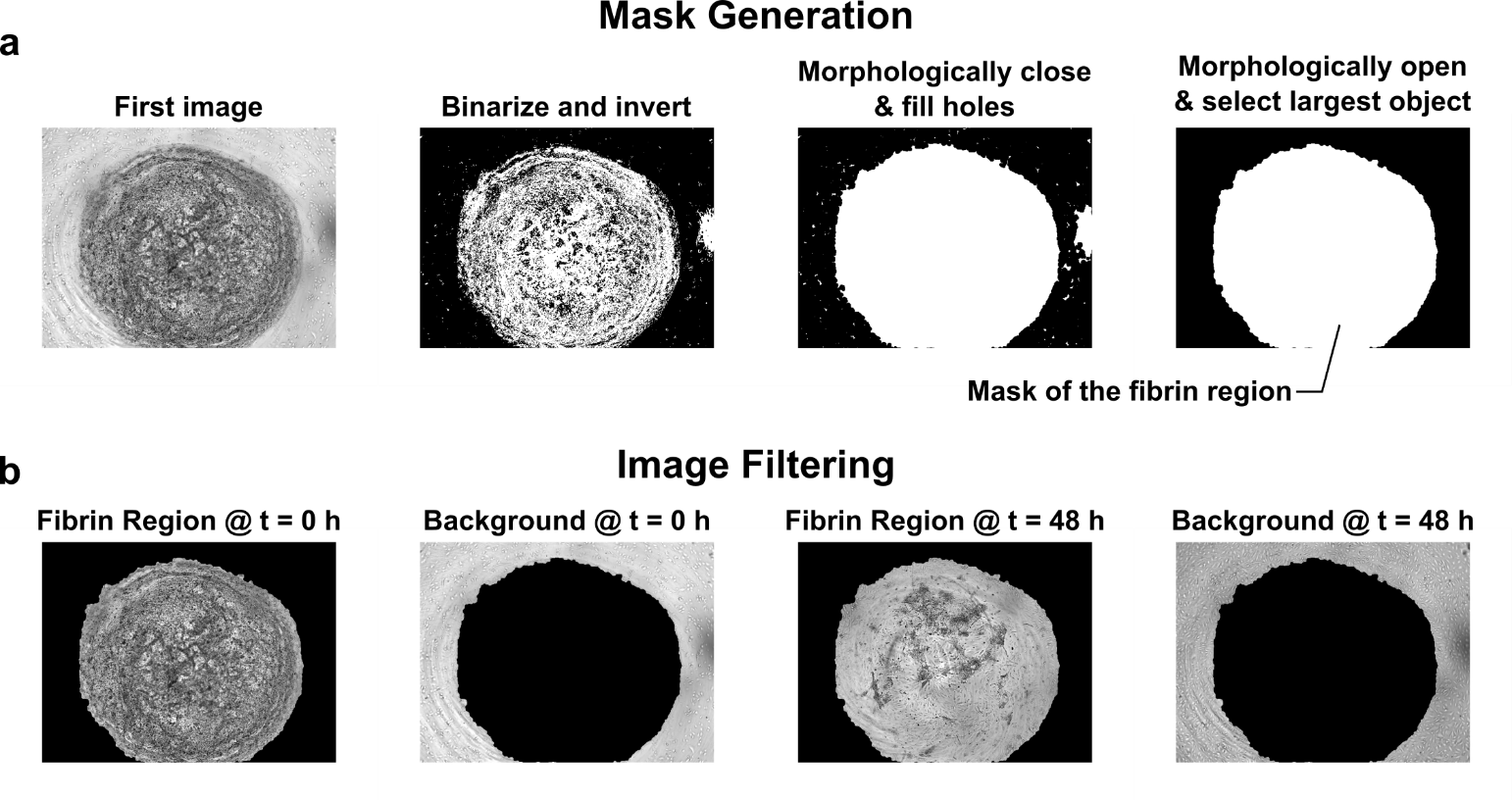


**Supplementary Figure S1.** (**a**) Morphological image processing is used to first generate a mask of the fibrin region. (**b**) This mask allows the fibrin region to be separated from the image background. Pixel intensity changes in only the fibrin region can now be calculated to eliminate noise from the background.


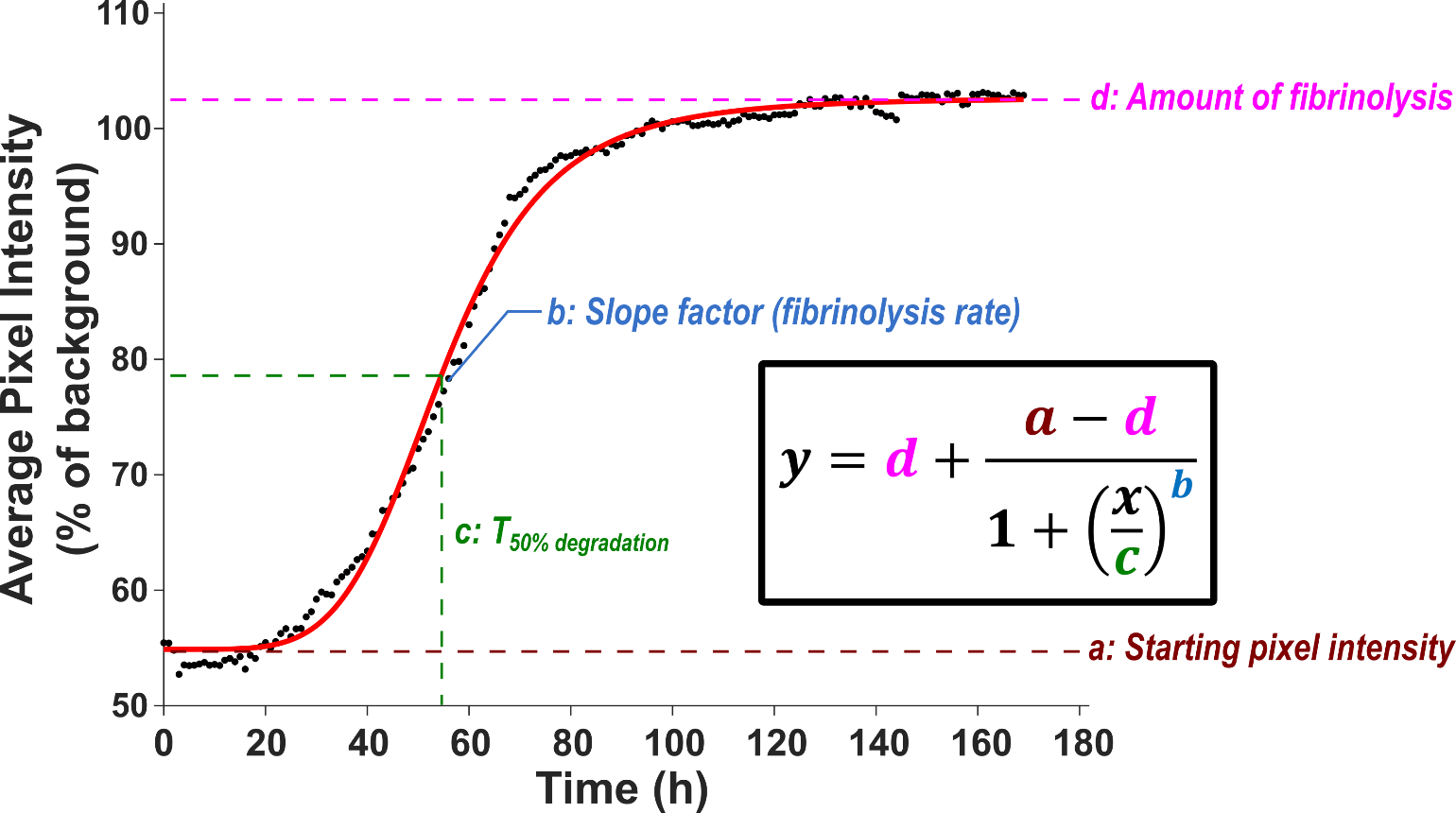


**Supplementary Figure S2.** Average pixel intensity over time can be fit to a 4-parameter logistic curve. Parameter c represents the time it takes to reach 50% of the total change in average pixel intensity. Parameter c is an indicator of fibrinolysis speed and referred to as fibrinolysis time.


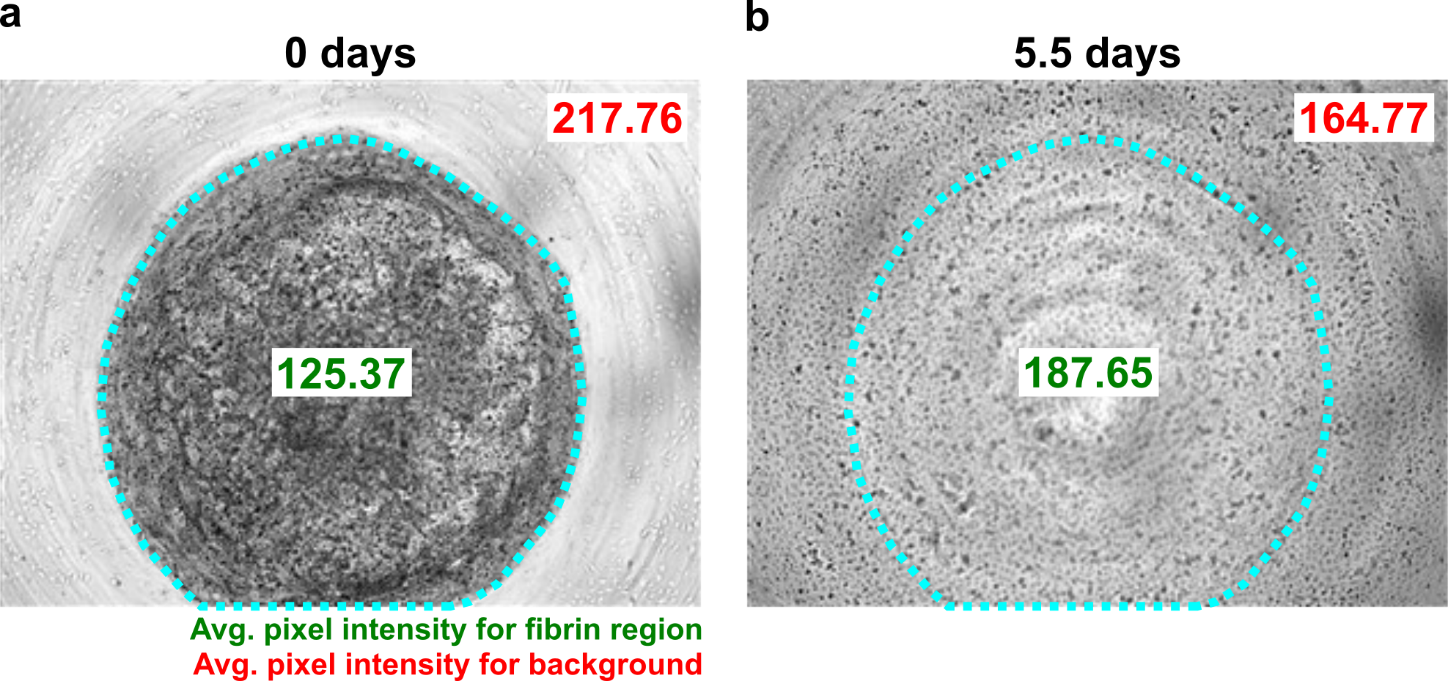


**Supplementary Figure S3.** (**a**) Images of the fibrin micro-clots are darker in the fibrin region than the background at the day 0. (**b**) After complete fibrinolysis, the fibrin region is brighter than the background. This characteristic of the Incucyte images causes the normalized average pixel intensity to reach values over 100%.


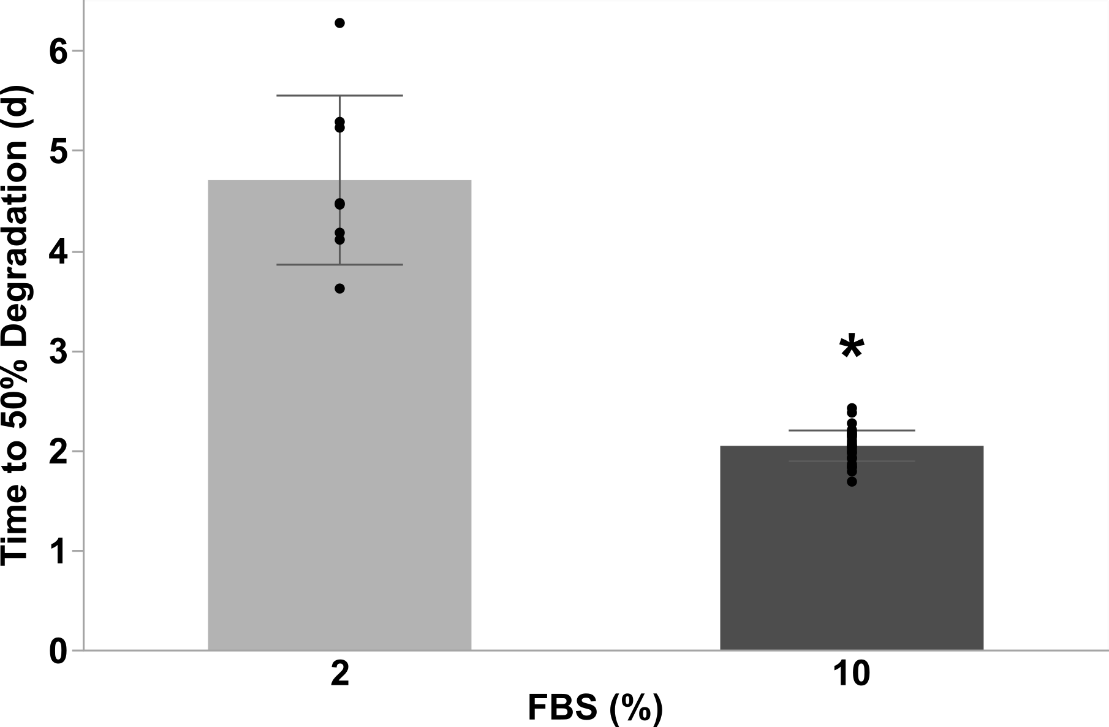


**Supplementary Figure S4.** Higher percentages of serum result in significantly faster fibrinolysis times. N = 8 for 2% serum conditon. N = 40 for the 10% FBS condition compiled from 5 independent experiments. Data is shown as mean ± standard deviation.

**Supplementary Figure S5.** Percent cytotoxicity based on LDH-Glo assay remains less than 25% for the entire assay duration. For LPS conditions, cells were pre-treated with LPS for 24 h prior to clot-loading. N = 8 for each condition. Statistical significance was determined using a Student’s t-test between the LPS and non-LPS conditions of the same timepoint. P-value: < 0.01 (**), < 0.001 (***), < 0.0001 (****).


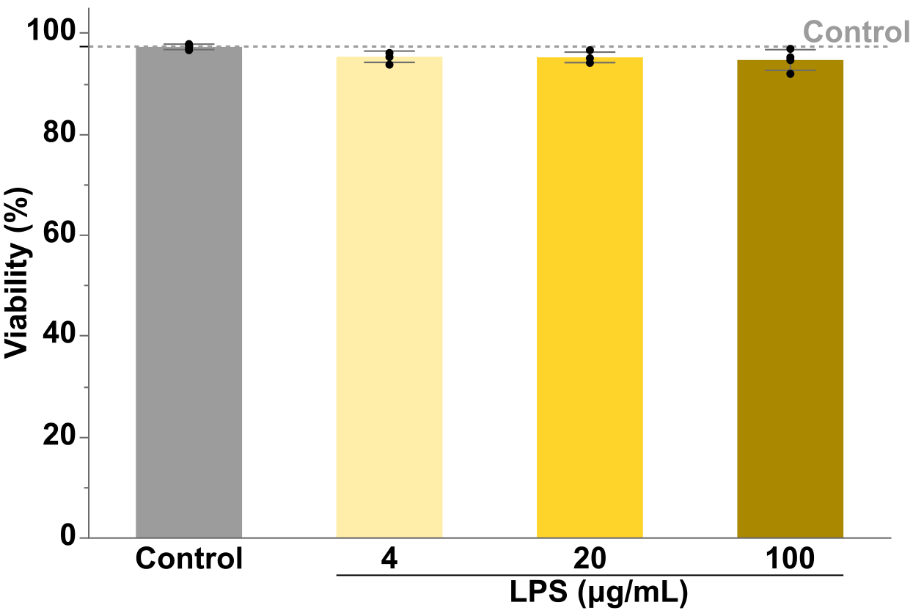


**Supplementary Figure S6.** Results of LIVE/DEAD staining with LPS treatment for 24 hours. No significant difference in cell viability was observed with LPS treatment. N = 4 for all conditions.


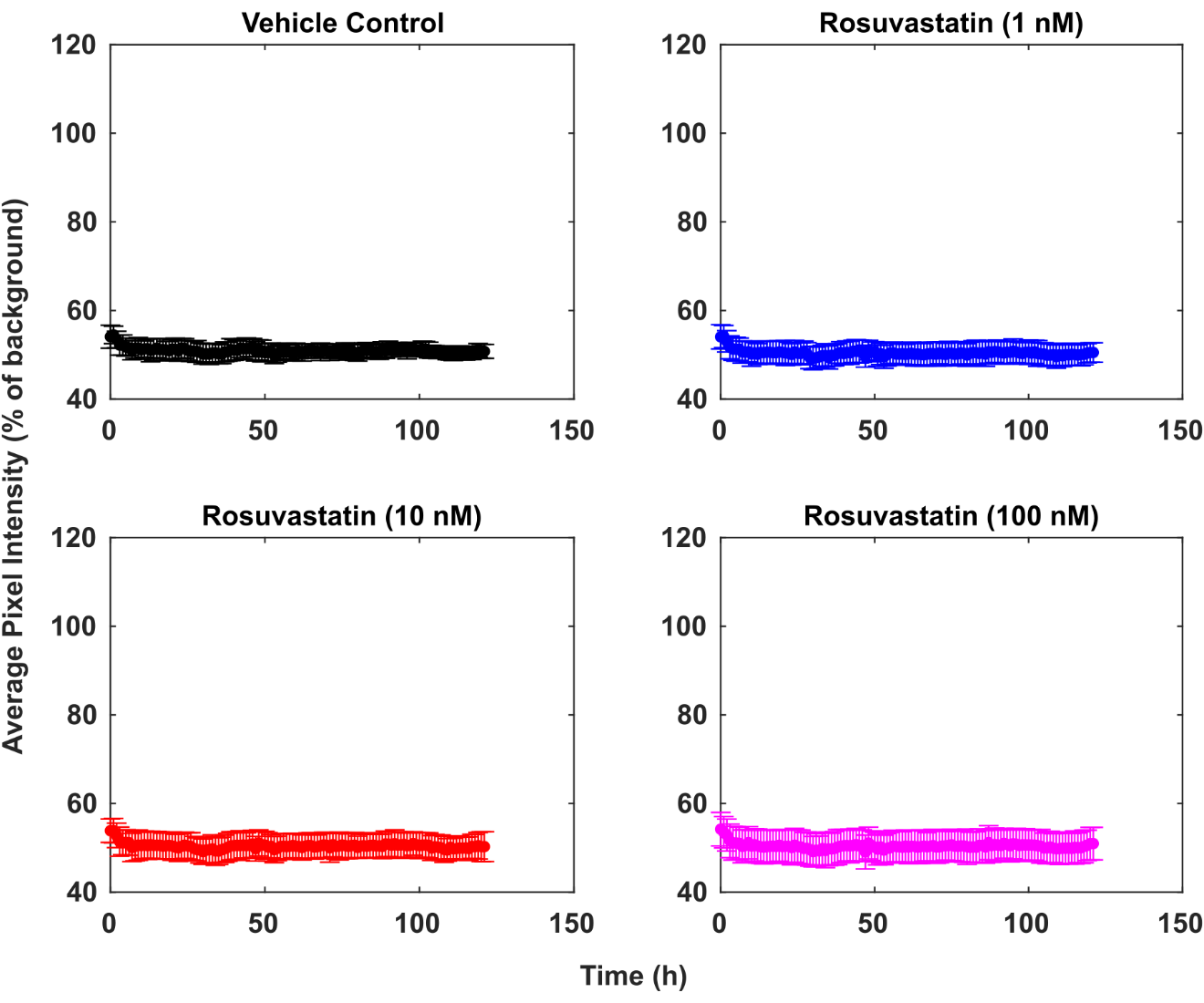


**Supplementary Figure S7**. Without HUVECs present, rosuvastatin does not enhance fibrinolysis. Acellular fibrin micro-clots were stable and did not degrade over the course of 5 days. N = 8 for all conditions and data is shown as mean ± standard deviation.


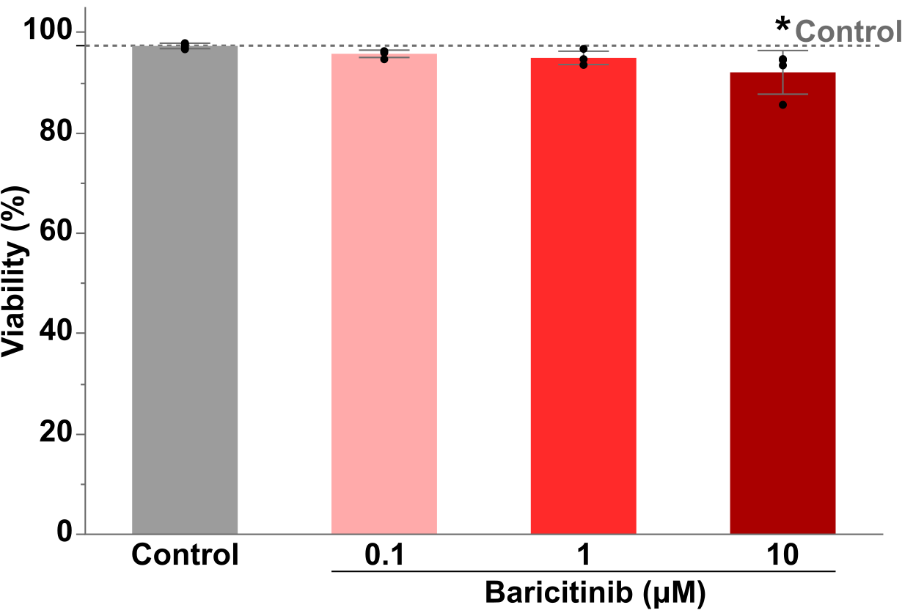


**Supplementary Figure S8**. Results of LIVE/DEAD staining with baricitinib treatment for 24 hours. The highest baricitinib concentration of 10 μM significantly decreases cell viability (mean viability = 92.11%). N = 4 for all conditions (* P < 0.05 by ANOVA with post-hoc Tukey’s test).


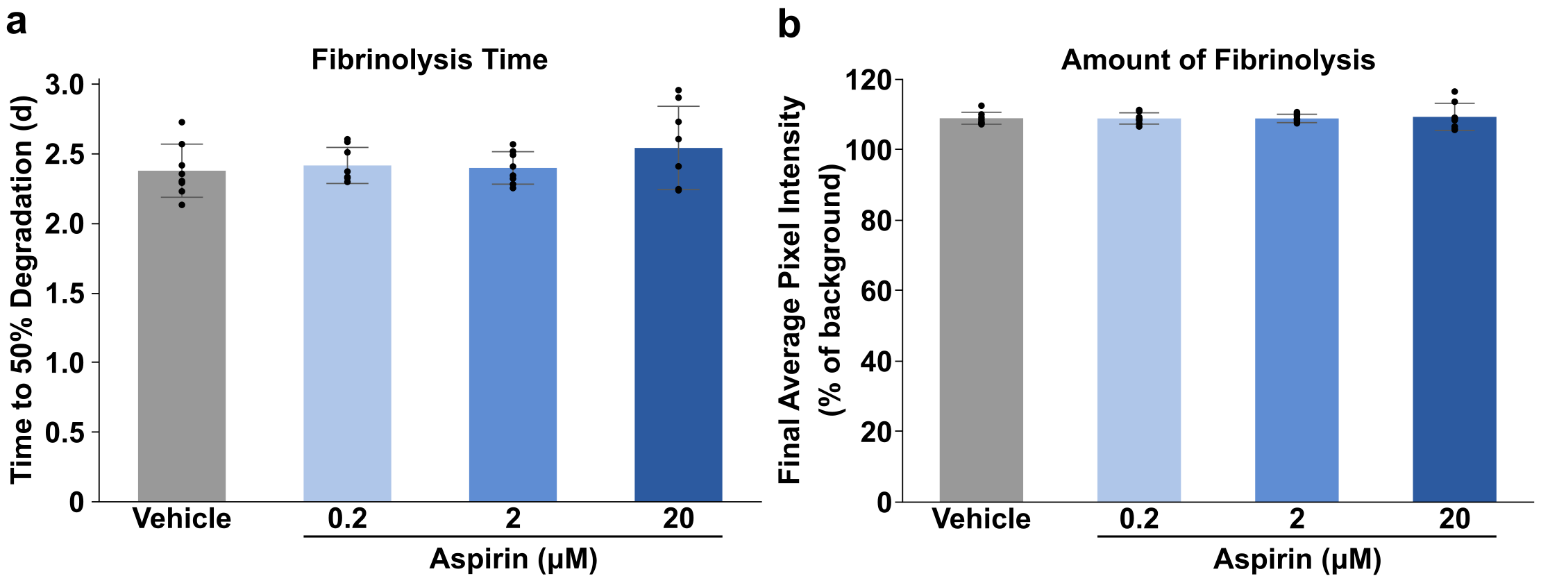


**Supplementary Figure S9**. Aspirin treatment shows no effect on endothelial cell-mediated fibrinolysis. (**a**) Fibrinolysis time and (**b**) amount of fibrinolysis at increasing aspirin concentrations. All data is shown as mean ± standard deviation. N = 8 for all conditions.


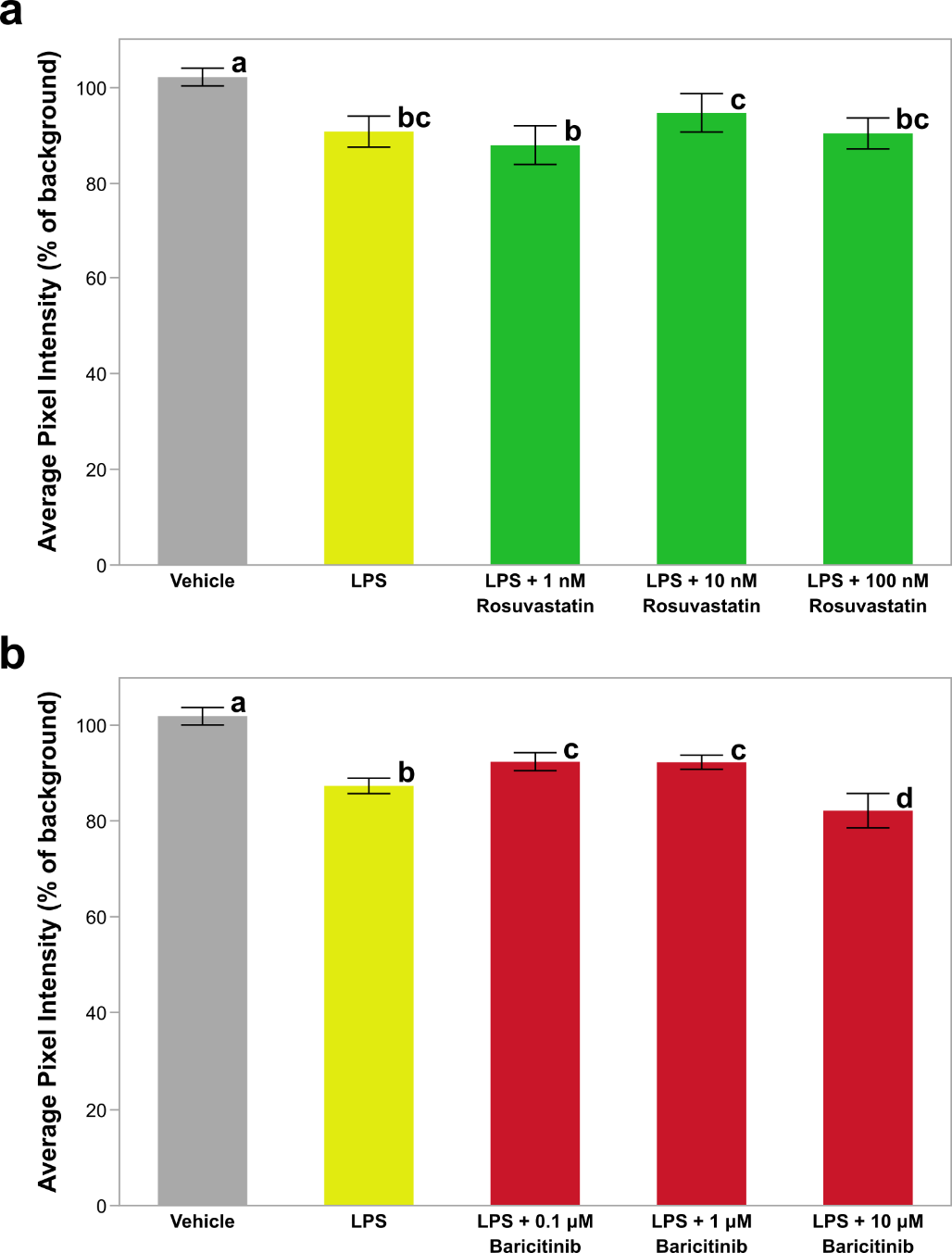


**Supplementary Figure S10.** Average pixel intensity at day 7 for endothelial cells pre-treated with LPS for 24 h before drug addition. (**a**) Rosuvastatin treatment does not significantly impact fibrinolysis in comparison to the LPS control. Conditions not connected by the same letter are statistically significant (P < 0.05 by ANOVA with post-hoc Tukey’s test). N = 5 for all conditions. (**b**) Baricitinib treatment at 0.1 and 1 µM significantly increases the average pixel intensity at day 7. Conditions not connected by the same letter are statistically significant (P < 0.05 by ANOVA with post-hoc Tukey’s test). N = 8 for all conditions.
